# Proinflammatory and Th2 Cytokines Regulate the High Affinity IgE Receptor (FcεRI) and IgE-Dependant Activation of Human Airway Smooth Muscle Cells

**DOI:** 10.1371/journal.pone.0006153

**Published:** 2009-07-07

**Authors:** Naresh Singh Redhu, Ali Saleh, Lianyu Shan, William T. Gerthoffer, Sam K. Kung, Andrew J. Halayko, Bouchaib Lamkhioued, Abdelilah S. Gounni

**Affiliations:** 1 Department of Immunology, Section of Respiratory Diseases, University of Manitoba, Winnipeg, Manitoba, Canada; 2 Department of Biochemistry & Molecular Biology, College of Medicine, University of South Alabama, Mobile, Alabama, United States of America; 3 Department of Physiology, Section of Respiratory Diseases, University of Manitoba, Winnipeg, Manitoba, Canada; 4 Laboratoire d'Immunologie et Microbiologie, UFR de Pharmacie de Reims ‘EA4303, IFR53’ Université de Reims Champagne-Ardenne, Reims, France; LMU University of Munich, Germany

## Abstract

**Background:**

The high affinity IgE receptor (FcεRI) is a crucial structure for IgE-mediated allergic reactions. We have previously demonstrated that human airway smooth muscle (ASM) cells express the tetrameric (αβγ2) FcεRI, and its activation leads to marked transient increases in intracellular Ca^2+^ concentration, release of Th-2 cytokines and eotaxin-1/CCL11. Therefore, it was of utmost importance to delineate the factors regulating the expression of FcεRI in human (ASM) cells.

**Methodology/Principal Findings:**

Incubation of human bronchial and tracheal smooth muscle (B/TSM) cells with TNF-α, IL-1β or IL-4 resulted in a significant increase in FcεRI-α chain mRNA expression (p<0.05); and TNF-α, IL-4 enhanced the FcεRI-α protein expression compared to the unstimulated control at 24, 72 hrs after stimulation. Interestingly, among all other cytokines, only TNF-α upregulated the FcεRI-γ mRNA expression. FcεRI-γ protein expression remained unchanged despite the nature of stimulation. Of note, as a functional consequence of FcεRI upregulation, TNF-α pre-sensitization of B/TSM potentially augmented the CC (eotaxin-1/CCL11 and RANTES/CCL5, but not TARC/CCL17) and CXC (IL-8/CXCL8, IP-10/CXCL10) chemokines release following IgE stimulation (p<0.05, n = 3). Furthermore, IgE sensitization of B/TSM cells significantly enhanced the transcription of selective CC and CXC chemokines at promoter level compared to control, which was abolished by Lentivirus-mediated silencing of Syk expression.

**Conclusions/Significance:**

Our data depict a critical role of B/TSM in allergic airway inflammation via potentially novel mechanisms involving proinflammatory, Th2 cytokines and IgE/FcεRI complex.

## Introduction

Airway inflammation has been considered as a critical factor in the pathogenesis of allergic asthma, often associated with bronchial hyperresponsiveness and is correlated with disease severity [1]. The inflammation is mainly due to an increased number of activated T lymphocytes, mast cells, eosinophils, and neutrophils within the airway lumen and bronchial submucosa [1], [2]. The CD4^+^ T cells have been demonstrated as the predominant cell type involved in the regulation of airway inflammation through the expression of T helper 2 (Th2) cytokines [2].

Besides these prototype inflammatory cells, however, airway smooth muscle (ASM) cells have been described recently as a rich source of proinflammatory cytokines, chemokines, and growth factors; and have been considered as key inflammatory determinants of asthma pertaining to their ability to contract in response to these mediators [3]. ASM cells can contribute directly to the pathogenesis of asthma by expressing cell adhesion and co-stimulatory molecules and by secreting multiple proinflammatory cytokines and chemokines that may perpetuate airway inflammation and the development of airway remodeling *in vivo* [4]. Therefore, it is now widely accepted that ASM actively participates in the pathogenesis of allergic asthma, by virtue of its role in airway inflammation, airway hyperresponsiveness and airway remodeling.

Several studies have led to novel insights into immune processes and the role of IgE in atopic diseases such as asthma [5]. Bronchial hyperresponsiveness has been shown to be associated with increased serum IgE levels and could be transferred from asthmatic to non-asthmatic subjects by IgE-rich serum administration [5]. The biological activities of IgE are mediated through the high affinity (FcεRI) and the low affinity (FcεRII or CD23) IgE receptors [6], [7]. Initially discovered on mast cells and basophils which are involved in cellular degranulation, FcεRI has been shown to be expressed by many inflammatory cells including Langerhans cells, dendritic cells, monocytes, eosinophils, platelets, neutrophils from allergic asthmatics, and bronchial epithelial cells [7], [8]. Very recently, FcεRI expression was detected in pinealocytes, the melatonin-secreting cells of the pineal gland, where it is proposed to provide a missing link between the neuroendocrine and the immune system in context of the neural control of allergic disorders [9].

Classically, TNF-α and IL-1β are prototype pro-inflammatory cytokines, which have been shown to play a central role in lung inflammation both in animal models and allergic patients [10], [11], [12], [13]. Th-2 cytokines are also known to play a critical role in pathogenesis of allergic disorders, and have been investigated extensively in strategies targeting respiratory diseases [14]. In particular, IL-4, in combination with IL-13 is known to induce Th-2 humoral immune responses leading to IgE production by B-cells [6].

We have previously demonstrated that human ASM (HASM) cells express the tetrameric (αβγ2) FcεRI and its activation leads to transient increases in intracellular Ca2^+^ concentration, and release of IL-4, IL-5, IL-13 and the CC chemokine eotaxin-1/CCL11 [15]. Taking into account the proinflammatory and Th-2 cytokine milieu in allergic inflammation and expression of FcεRI on ASM, it was imperative to examine the factors regulating the expression of FcεRI in human ASM cells. FcεRI-α-chain, a member of the immunoglobulin superfamily, contains the binding site for its ligand (IgE) while both β and γ chains are responsible for the downstream signal propagation through the phosphorylation of their immunoreceptor tyrosine-based activation motif (ITAM) [7].

Here we demonstrate that TNF-α, IL-1β, and IL-4 induce the FcεRI-α mRNA and protein expression in human bronchial/tracheal smooth muscle (B/TSM) cells. The functional studies demonstrated that the IgE stimulation of cytokine pre-sensitized B/TSM cells significantly augmented the selective CC and CXC chemokine expression. Interestingly, Lentivirus mediated spleen tyrosine kinase (Syk) silencing abrogated the IgE sensitization-induced transcription of selective CC and CXC chemokines at promoter level. Cumulatively, these results suggest the potentially novel mechanisms of FcεRI regulation, portraying the critical role of B/TSM associated IgE/FcεRI complex in allergic airway inflammation.

## Materials and Methods

### Ethics statement

All the experimental procedures were approved by the Human Research Ethics Board of the University of Manitoba, Winnipeg, Manitoba, Canada. Written informed consent for ASM harvesting was obtained from all patients.

### Reagents

Recombinant human TNF-α, IL-1β and IL-4 were purchased from R&D Systems (Minneapolis, MN, USA). Recombinant human IgE was obtained from Diatec (BioPorto Diagnostics A/S, Denmark). Murine anti-human FcεRIα-chain mAb, 15/1 [16], was kindly donated by Dr. Franz Kricek (NOVARTIS Institute for Biomedical Research GmbH & Co KG, Vienna, Austria). Fetal bovine serum (FBS), sodium pyruvate, trypsin were purchased from HyClone (Logan, UT, USA). 100X L-glutamine, DMEM, Ham's F-12, trypsin-EDTA, and antibiotics (penicillin, streptomycin) were purchased from Invitrogen Canada Inc. (Burlington, ON, Canada). Plasmids encoding luciferase reporter driven by respective wild-type chemokine promoters were kindly gifted (pGL3-EO2 eotaxin-1/CCL11 pr by Dr. Jutta Horejs-Hoeck, Institute for Chemistry and Biochemistry, Salzburg, Austria; pUHC13-3-IL-8pr-wild type by Dr. Michael Kracht, Medical School Hannover, Hannover, Germany; pGL3-RANTES-Luc wild-type by Dr. Akira Andoh, Shiga University of Medical Science, Seta-Tukinowa, Otsu 520-2192, Japan; pGL3-IP-10 (-533) by Dr. Daniel A Muruve, University of Calgary, Calgary, Alberta, Canada). Unless stated otherwise, all other reagents were obtained from Sigma-Aldrich Canada Ltd. (Oakville, ON, Canada).

### Preparation of Human Bronchial/Tracheal Smooth Muscle (B/TSM) Cells

Three sources of human airway smooth muscle cells were used. Both hTERT immortalized and primary human bronchial smooth muscle (HBSM) cells were prepared as described by us previously [18], [19]. Primary human tracheal smooth muscle (HTSM) cells were obtained from macroscopically healthy segments of the trachea during post-mortem in Respiratory Hospital at Health Sciences Centre, Winnipeg, MB, Canada, and were isolated, cultured as we used for primary HBSM cells [15], [18], [19]. At confluence, primary B/TSM cells exhibited spindle morphology and a hill-and-valley pattern that is characteristic of smooth muscle in culture. Moreover, B/TSM cells at confluence retain smooth muscle-specific actin, SM22, and calponin protein expression and mobilize intracellular Ca2+ in response to acetylcholine, a physiologically relevant contractile agonist [19]. In all the experiments, primary B/TSM cells were used at passages 2–5, and hTERT cells at passages 10–17.

### Cell stimulation

Sub-confluent B/TSM cells were growth arrested and synchronized by serum deprivation for 48 h in Ham's F-12 medium containing 5 µg/ml human recombinant insulin, 5 µg/ml human transferrin, 5 ng/ml selenium, and antibiotics (100 U/ml penicillin and 100 µg/ml streptomycin). Cells were then stimulated in fresh FBS-free medium containing human recombinant IL-1β (10 ng/ml), TNF-α (10 ng/ml), IL-4 (10 ng/ml), IgE (1 or 10 µg/ml) or vehicle (medium alone) for time periods specific to experiments, as mentioned below.

### RNA isolation and RT-PCR

Serum-deprived confluent B/TSM cultures were harvested, and total cellular RNA was extracted using TRIzol® method (Invitrogen Canada Inc., Burlington, ON). Reverse transcription was performed by using 2 µg of total RNA in a first-strand cDNA synthesis reaction with High Capacity cDNA Reverse transcriptase kit as recommended by the supplier (Applied Biosystems, Foster City, CA, USA). Oligonucleotide primers were synthesized on the basis of the entire coding region of human FcεRI-α (GenBank accession no. NM 002001.2) as follows: Forward primer 5′-CTCCATTACAAATGCCACAGTTG-3′ and Reverse primer 5′-CACGCGGAGCTTTTATTACAGTA-3′; and for human FcεRI-γ (GenBank accession no. NM004106) were: Forward primer, 5′-CCA GCA GTG GTC TTG CTC TTA C-3′ and reverse primer, 5′-GCA TGC AGG CAT ATG TGA TGC C-3′. Primers for human housekeeping gene, glyceraldhyde-3-phosphate dehydrogenase (GAPDH) are forward primer 5′-AGCAATGCCTCCTGCACCACCAAC-3′ and reverse primer 5′-CCGGAGGGGCCATCCACAGTCT-3′. The PCR (FcεRI-α, 35 cycles; FcεRI-γ, 30 cycles; GAPDH, 25 cycles) was conducted in a thermal cycler ‘Mastercycler’ (Eppendorf Canada, Mississauga, ON). Each cycle included denaturation (94°C, 1 min), annealing (FcεRI-α, 59°C, 1 min; FcεRI-γ, 64°C, 1 min and; GAPDH, 55°C, 1 min), and extension (72°C, 1 min 30 s). The initial denaturation period was 5 min, and the final extension was 10 min. The size of the amplified FcεRI-α, -γ and GAPDH fragment was 116 bp, 338 bp and 137 bp, respectively. GAPDH was amplified as internal control. Amplified products were analyzed by DNA gel electrophoresis in 1.5% agarose and visualized by ethidium bromide staining under ultraviolet illumination. The specificity of the amplified bands was confirmed by nucleic acid sequencing (data not shown).

### Real-time RT-PCR analysis

The FcεRI-α and GAPDH standards were prepared using PCR-amplified cDNA from a human basophilic cell line (KU812, ATCC® # CRL-2099™). PCR products were isolated from 2% w/v agarose gel using QIAEX II Agarose Gel Extraction kit (Qiagen Inc., Mississauga, ON, Canada). The primers used were same as we used for RT-PCR, as mentioned above. The amount of extracted cDNA was quantified by spectrophotometry and expressed as copy number. A serial dilution was used to generate each standard curve. Real-time quantitative PCR was carried out by ABI 7500 Real-Time PCR System and analyzed by 7500 System SDS software version 1.3.1 (Applied Biosystems, Foster City, CA, USA), following manufacturer's instructions. Product specificity was determined by melting curve analysis and by visualization of PCR products on agarose gels. Calculation of the relative amount of each cDNA species was performed according to standard protocols. Briefly, the amplification of FcεRI-α gene in stimulated cells was calculated first as the copy number ratio of FcεRI-α to GAPDH, and then expressed as normalized values of fold increase over the value obtained with unstimulated (control) cells.

### Immunoprecipitation and Western blot

B/TSM FcεRIα-chain protein expression was analyzed by immunoprecipitation and Western blotting with minor modifications from the protocol described earlier [8]. Briefly, B/TSM cells were lysed for 30 min 4°C in NP-40 lysis buffer supplemented with a cocktail of protease inhibitors (2 mM sodium orthovanadate, 1 mM phenyl-methylsulfonylfluoride, 10 µg/ml leupeptin, 0.15 units/ml approtinin, 1 µg/ml pepstatin A) (Sigma-Aldrich) and centrifuged for 20 min to remove nuclei. Cell lysates from B/TSM or basophilic cell line KU812 (positive control) were pre-cleared with protein G sepharose-coated beads (Amersham-Pharmacia) for 2 h at 4°C in a rotating mixer, followed by incubation with protein G sepharose-coated beads conjugated with 2 µg/ml of murine anti-human FcεRIα mAb 15/1 or isotype mouse IgG1 monoclonal antibody (MOPC 21) for 16 h at 4°C. Immuno-complexes were then pelleted by centrifugation and washed six times with the wash buffer (PBS/1% NP40). For immunoblotting, samples were separated on SDS polyacrylamide gel (11–13%) and electro-transferred onto PVDF membrane (Millipore, Mississauga, ON). The membrane was blocked at RT for 2 hrs with 5% Blotto, (Santa Cruz Biotechnology, CA, USA), incubated with rabbit anti-human FcεRIα-chain polyclonal Ab (Upstate Biotechnology, Inc., Lake Placid, NY) (1 µg/ml) at room temperature for 2 h, followed by secondary antibody HRP-goat anti-rabbit IgG (H+L) prepared in TBST (1∶5000). The blots were developed by enhanced chemiluminescence as recommended by the supplier (Amersham Pharmacia, ON). A band non-specific to FcεRI in the same gel was used as loading control. On the other hand, 15 µg of B/TSM and KU812 cell lysates were directly loaded onto 14% SDS polyacrylamide gel (11–13%) and electro-transferred onto PVDF membrane. The membrane was blocked at RT for 2 hrs with 5% Blotto, incubated with goat anti-human FcεRI-γ polyclonal Ab (K-16 clone) (Santa Cruz, CA) prepared in TBST (1∶500) at RT for 2 h, followed by secondary antibody HRP-rabbit anti-goat IgG whole molecule (Sigma-Aldrich) prepared in TBST (1∶5000), and the blots were developed as described above. After stripping, the blots were probed for GAPDH and used as a loading control. The corresponding values of pre-stained protein molecular weight marker were scaled to the FcεRI-α and -γ protein bands. The intensity of FcεRI-γ bands was determined by using AlphaEase FC software version 3.1.2 relative to control loading levels.

### CC and CXC Chemokines ELISA from cytokine pre-sensitized B/TSM cell supernatants

In order to study the functional significance of cytokine stimulated FcεRIα-chain upregulation, primary human B/TSM cells were grown until 65% confluency in 12-well culture plates and 48 h serum-deprived cells were then stimulated for 48 h with recombinant human IL-1β, TNF-α, IL-4 (10 ng/ml each) or vehicle (medium alone). The supernatants were removed and cells were washed twice with serum-free Ham's F12 media. Thereafter, the cells in each treatment group were either left unstimulated (medium alone) or stimulated with mIgG1 (1 µg/ml) or recombinant human IgE (1 µg/ml) for 24 h under the similar culture conditions. The culture supernatants were then collected, centrifuged at 1200 rpm for 7 min at 4°C to remove cellular debris and stored at −80°C until future use. Immunoreactive eotaxin-1/CCL11, IL-8/CXCL8, IP-10/CXCL10 (10 kDa interferon-gamma-induced protein), RANTES/CCL5 (Regulated upon Activation, Normal T-cell Expressed, and Secreted), and TARC/CCL17 (Thymus and activation-regulated chemokine) released into B/TSM culture supernatants were quantified using ELISA with matched Abs according to basic laboratory protocol provided by the manufacturer (R&D Systems, Minneapolis, MN, USA). Chemokine proteins were quantified in reference to serial dilutions of recombinant standards falling within the linear part of the standard curve for each specific chemokine sample measured. The sensitivity limits of these chemokine assays are 5 pg/ml for eotaxin-1/CCL11, 3 pg/ml for IL-8/CXCL8, 7.8 pg/ml for IP-10/CXCL10, 7.8 pg/ml for RANTES/CCL5 and 7.8 pg/ml for TARC/CCL17. Each data point represents readings from three separate assays.

### Lentiviral vector transduction in B/TSM cells

For short-hairpin RNA (shRNA)-induced gene silencing studies, pseudotyped lentiviral vector (clone Id: V2LHS_153702) expressing specific Syk shRNA was obtained from Open-Biosystems (Huntsville, AL). 293T cells used for virus production and titration, were cultured in Iscove's modified Dulbecco's medium (HyClone, Logan, UT) supplemented with 10% fetal bovine serum (FBS), and 1% penicillin/streptomycin/glutamate (PSG) (Gibco, Grand Island, NY). Lentivirus were generated using 293T cell lines and viral titer was determined by counting the puromycin resistant colonies, as described elsewhere [21]. A control shRNA unrelated to Syk sequence (scramble shRNA) was used as a transduction control. For silencing Syk protein expression, B/TSM cells were transduced at a multiplicity of infection (MOI) of 10 in the presence of polybrene (8 µg/ml). In brief, cells were exposed to recombinant lentivirus for 2 hr at 37°C, medium replaced and cultured for additional 72 hrs. Transduced cells were selected with puromycin. The average transduction efficiency was more than 95% as determined by FACS using the turbo-green fluorescent protein (tGFP) as the marker for cell sorting. Viability of the transduced cells undergoing experiment was >98% as assessed by trypan blue dye after completion of the experiment.

### Luciferase reporter constructs and cell transfection

Normal, Syk-silenced, and scramble-shRNA-transduced B/TSM cells (4×10^4^) were plated into 12-well culture plates in fresh complete DMEM. At 50–70% confluency, cells were transfected with wild-type plasmid constructs containing promoters for human eotaxin-1/CCL11, IL-8/CXCL8, IP-10/CXCL10 or RANTES/CCL5. Transient transfection of B/TSM cells was performed using ExGen 500 *in vitro* transfection reagent (MBI Fermentas, ON, Canada) according to the manufacturer's instructions. In each well, 1.6 µg of wild-type chemokine promoter DNA and 0.4 µg of *Renilla* luciferase reporter vector-pRL-TK (Promega, Madison, WI) were co-transfected for 24 h. The medium was changed and cells were washed and stimulated with human IgE (10 µg/ml), IL-1β (10 ng/ml) or mouse IgG1 (mIgG1-MOPC21) (10 µg/ml). Since IL-1β is known to induce multiple cytokines/chemokines gene expression in human ASM cells [22], [23], [24], [25], it was used as a positive control for promoter activity assays. The luciferase activity was measured by the Dual-Luciferase Assay System kit (Promega, Madison, WI) using a luminometer (model LB9501; Berthold Bad Wildbad, Germany). Briefly, 20 µl of cell lysate was mixed with 100 µl of Luciferase Assay Reagent II and firefly luciferase activity was first recorded. Then, 100 µl of Stop-and-Glo Reagent was added, and *Renilla* luciferase activity was measured. All values were normalized to *Renilla* luciferase activity and expressed relative to the control transfected non-stimulated cells.

### Statistical analysis

All the data were obtained from experiments performed three or more times. Statistical analysis was performed by using GraphPad Prism Software Version 3.02 for Windows (GraphPad Software Software, San Diego, CA, USA). Association between chemokine expression levels in the subgroups and cytokine stimulation effect on FcεRI expression were studied using Mann-Whitney U test. P values <0.05 were considered statistically significant.

## Results

### TNF-α, IL-1β, and IL-4 regulate the FcεRI-α chain mRNA Expression in Human B/TSM cells

**Figure 1:**
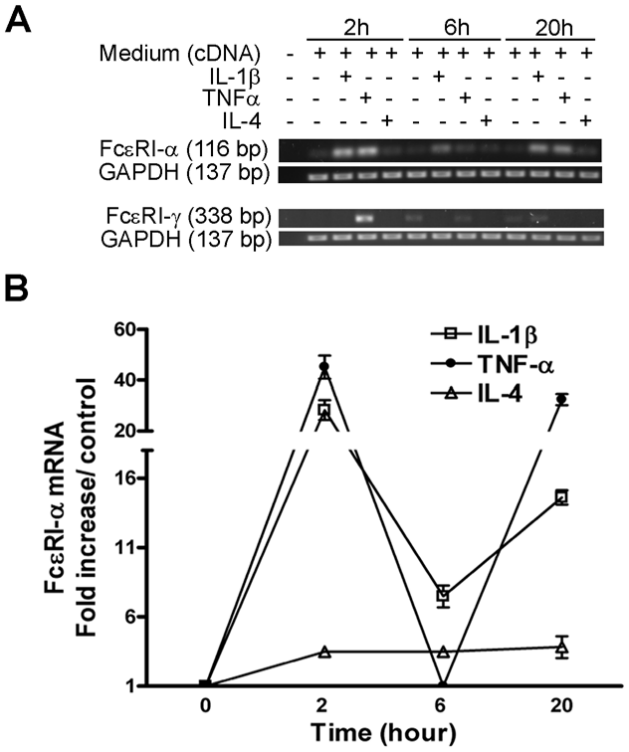
Proinflammatory and Th-2 cytokines upregulate the FcεRI mRNA expression in human B/TSM cells. Human B/TSM FcεRI-α and –γ mRNA expression was analysed by (A) RT-PCR and (B) by Real-time RT-PCR for FcεRI-α chain. GAPDH was used as internal control for (A) and to normalize the FcεRI-α copy number for (B) as described in *Materials and Methods*. Each data point (except for TNF-α at 6 h) represents a significant (p<0.05, n = 3) increase in copy no. over unstimulated control. P values were calculated using Mann Whitney U test.

We previously speculated the probable modulation of FcεRI expression in ASM cells by proinflammatory and Th2 cytokines [15], [26]. In the present study, human B/TSM cells stimulated with IL-1β, TNF-α, or IL-4 showed significantly enhanced FcεRI-α mRNA expression (Figure 1A). In contrast to cytokine stimulation, the basal FcεRI-α mRNA expression was uniform and unaffected by time of culture. FcεRI-α mRNA increased expression was then confirmed by quantitative real-time RT-PCR analysis. As shown in Figure 1B, B/TSMCs stimulated with TNF-α, and IL-1β upregulated the FcεRI-α transcript expression by 45.0±4.5-, and 28.2±3.8-fold, respectively, compared to unstimulated cells at 2 h. Interestingly, the mRNA expression was downregulated at 6 h but again gained peak at 20 h; whereas TNF-α inducing the maximum expression (32.4±2.1-fold). Notably, IL-4 stimulation steadily upregulated the FcεRI-α mRNA expression (2.44±0.28-fold, 2 h; 2.43±0.29-fold, 6 h; and 2.79±0.7-fold, 20 h) compared to the control in B/TSM cells (Figure 1B). On the other hand, the mRNA expression for FcεRI-γ chain was upregulated by TNFα only at 2 h (Figure 1A). Collectively, this data suggest that proinflammatory and Th-2 cytokines can potentially regulate the transcription of FcεRI in B/TSM cells.

### TNF-α, IL-4 upregulate the FcεRI-α protein expression in human B/TSMCs

**Figure 2:**
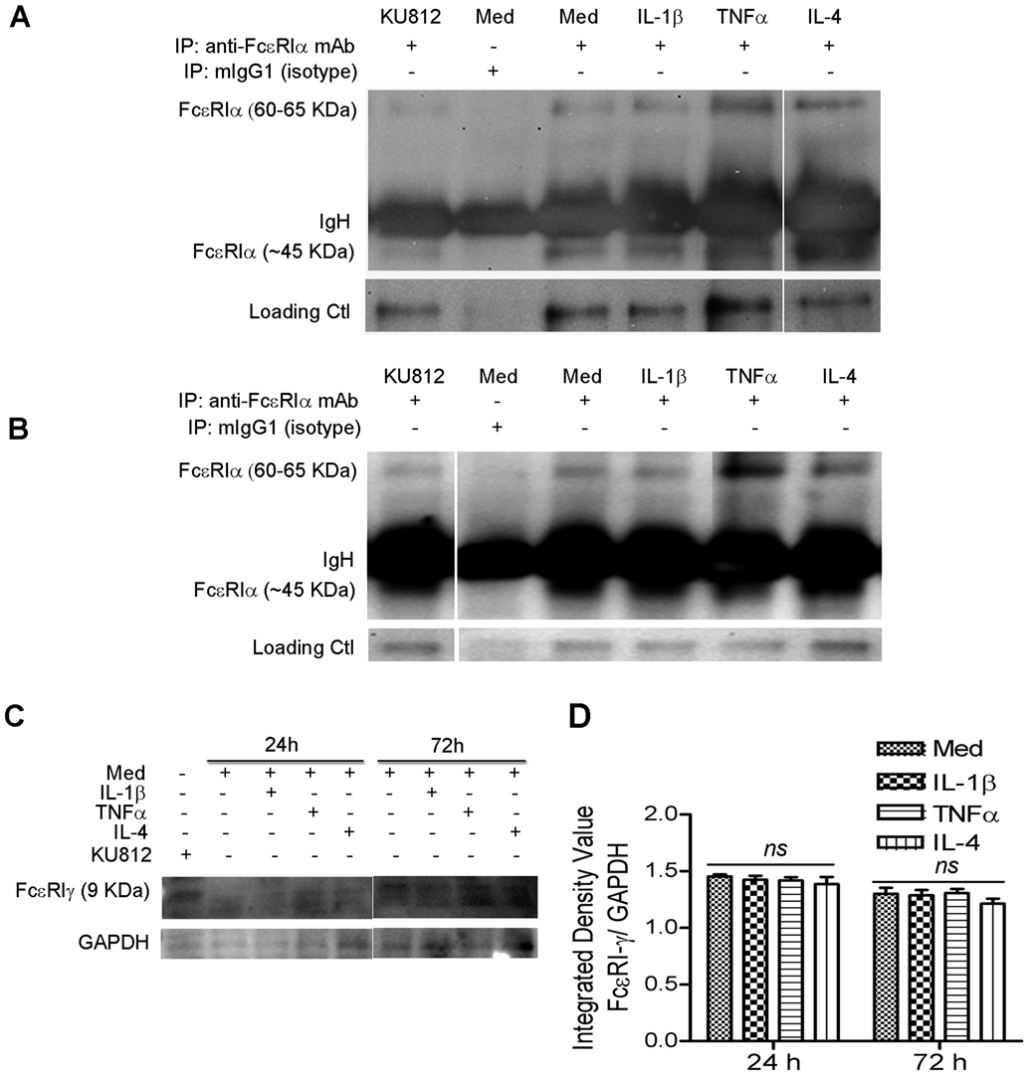
FcεRI-α protein expression (24, 72 h) is upregulated by proinflammatory and Th-2 cytokines. Two-day serum-deprived primary B/TSM cells were cultured in presence or absence of IL-1β, TNF-α or IL-4. FcεRI-α protein from (A) 24 h and (B) 72 h culture cell lysates was Immunoprecipitated (IP) followed by Western blotting, as described in *Materials and Methods* section. FcεRI-α protein was immunoprecipitated with either anti-FcεRIα mAb 15/1 or with isotype antibody mouse IgG1 (MOPC21) for negative control. Non-specific bands from the same gels were used as loading control. (C) FcεRI-γ protein was analyzed by Western blotting and (D) presented as the ratio of 9 KDa FcεRI-γ-specific protein bands intensity over GAPDH (as internal control). Human basophilic KU812 cells were used as a positive control. Figures represent three separate experiments. *ns*, non-significant (p>0.05).

To investigate the effect of IL-1β, TNF-α, and IL-4 stimulation on FcεRI-α and -γ protein expression, serum-deprived B/TSM cells were stimulated with TNF-α, IL-1β, or IL-4 and subjected to immunoprecipitation and Western blot. As demonstrated in Figure 2A, TNF-α and IL-4 stimulation enhanced the ∼45 KDa (referred to be the intracellular) [7], [8] FcεRI-α protein expression at 24 h compared to unstimulated or IL-1β-stimulated B/TSMCs. Immunoprecipitation from basophilic cell line KU812 also revealed a positive band at ∼45 KDa (Figure 2A).

We then explored the effect of chronic (72 h) cytokine stimulation on FcεRI-α protein expression. As shown in Figure 2B, TNF-α, and IL-4 stimulation for 72 h led to the upregulation of FcεRI-α protein in B/TSM cells. TNF-α stimulation remarkably augmented the ∼45 KDa intracellular and 60–65 KDa (referred to be as surface chain) [27] FcεRI-α protein compared to unstimulated B/TSM cells (Figure 2B). Chronic IL-4 stimulation also augmented the FcεRI-α protein expression, both for ∼45 KDa and ∼60–65 KDa bands (Figure 2A and B). In contrast to FcεRI-α and TNF-mediated FcεRI-γ mRNA upregulation, no significant upregulation in protein was observed (Figure 2C and D). As expected, KU812 cell lysate revealed both FcεRIα intracellular and surface, and FcεRI-γ protein bands and served as a positive control. Importantly, immunoprecipitation with isotype control antibody mIgG1-MOPC21 did not show any conspicuous bands of FcεRI-α either of ∼45 KDa or 60–65 KDa at 24 or 72 h stimulation. Collectively, our data demonstrate that TNF-α and IL-4 upregulate the FcεRI-α chain protein expression in B/TSM cells.

### TNF-α pre-sensitization augments the eotaxin-1/CCL11, IL-8/CXCL8, IP-10/CXCL10, RANTES/CCL5 but not TARC/CCL17 release in B/TSM following IgE stimulation

**Figure 3:**
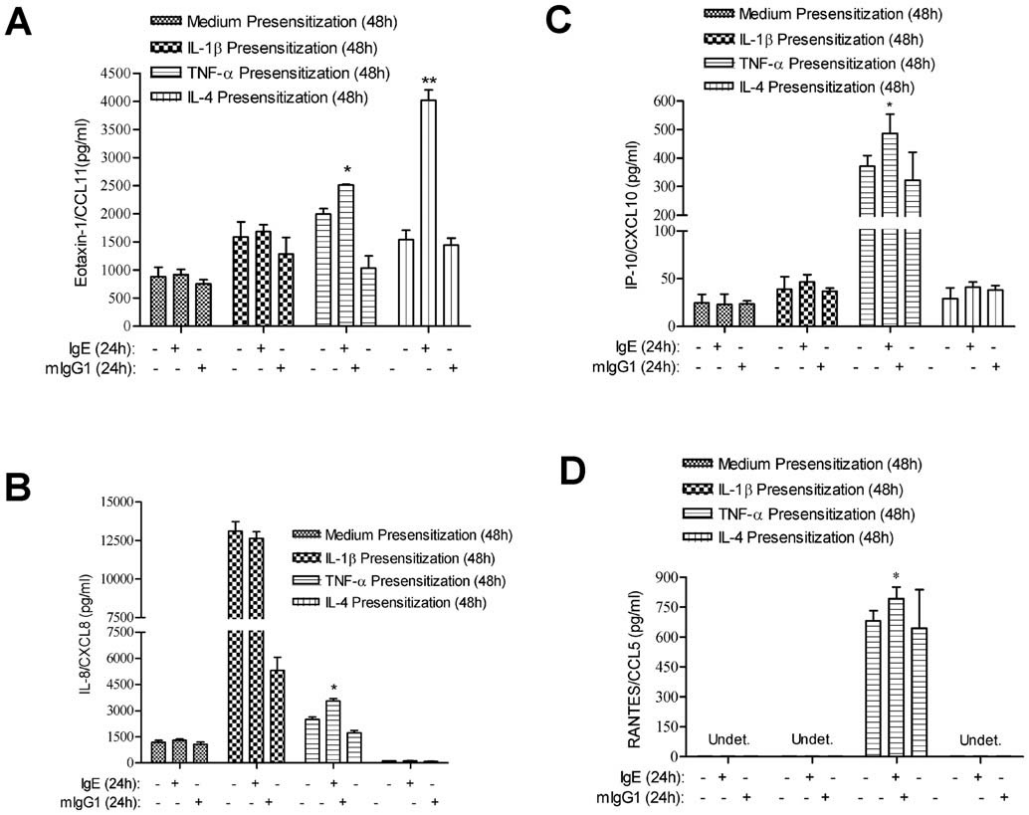
TNF-α pre-sensitization augments multiple CC and CXC chemokines release following IgE stimulation. Primary human B/TSM cells were cultured in presence of 10 ng/ml each of TNF-α, IL-1β, or IL-4 for 48 h. Cells were then washed twice, and stimulated with either IgE (1 µg/ml), mIgG1-MOPC21 (1 µg/ml) or left unstimulated (medium alone) for another 24 h. Culture supernatants were used for (A) eotaxin-1/CCL11, (B) IL-8/CXCL8, (C) IP-10/CXCL10, and (D) RANTES/CCL5 measurement by ELISA. Data represents mean±SD of three independent experiments performed under the same conditions. Mann-Whitney *U* test was performed to analyze the differences between the samples. *P<0.05, **P<0.01.

We then investigated the functional consequences of FcεRI-α protein upregulation by TNF-α, or IL-4 in B/TSM cells. Since we have previously demonstrated that IgE-dependent activation of B/TSM cells induces the CCL11 release [15], it was enticing to assess whether TNF-α or IL-4-mediated FcεRI-α protein upregulation followed by IgE stimulation can augment the chemokines release by B/TSM cells. Therefore, B/TSM cells were first sensitized with TNF-α, IL-1β, or IL-4 for 48 h, washed and then stimulated with IgE, mIgG1 (MOPC21) or left unstimulated for 24 h in fresh medium. CC (CCL11, CCL5, and CCL17) and CXC (CXCL8 and CXCL10) chemokines released in supernatants were then measured by ELISA. Since IL-1β did not induce a marked FcεRI-α protein expression (Figure 2A and B), it is plausible that it would not affect the subsequent chemokine release following IgE stimulation. Interestingly, TNF-α pre-sensitized B/TSM cells released significantly elevated (p<0.05, n = 3) levels of eotaxin-1/CCL11, IL-8/CXCL8, IP-10/CXCL10, and RANTES/CCL5 following IgE stimulation compared to IgE-unstimulated cells (Figure 3A, B, C and D). Moreover, IL-4 pre-sensitization followed by IgE stimulation also enhanced the eotaxin-1/CCL11 release significantly (p<0.01, n = 3, Figure 3A). We, however, did not see any significant change in TARC/CCL17 release in TNF-α, IL-1β, or IL-4 pre-sensitized and IgE stimulated B/TSM cells (data not shown), suggesting the selective nature of FcεRI activation-induced chemokine expression. These results, therefore, suggest that TNF-α, in particular, upregulates the FcεRI-α expression as considerably that subsequent stimulation of B/TSM with IgE engages and activates the FcεRI strongly, leading to enhanced selective chemokine release.

### IgE sensitization induces eotaxin-1/CCL11, IL-8/CXCL8, IP-10/CXCL10, and RANTES/CCL5 promoter activation in B/TSM cells

**Figure 4:**
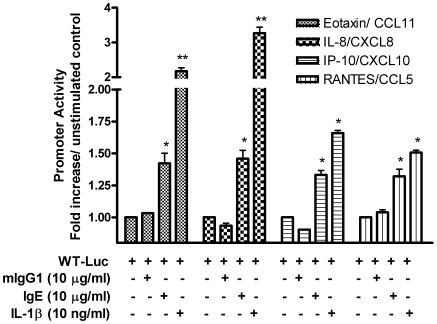
IgE-sensitization induces the chemokines promoter activity in human B/TSM cells. Cultured human B/TSM cells were transiently transfected with luciferase reporter constructs driven by wild-type promoters for human eotaxin-1/CCL11, IL-8/CXCL8, IP-10/CXCL10, and RANTES/CCL5; stimulated with mIgG1-MOPC21, IgE, or IL-1β; and respective chemokine promoter activity was measured as mentioned in *Materials and Methods*. Data were normalized according to the *Renilla* luciferase activity, and presented as fold-increase over unstimulated control. Data represents the mean±SD of three separate experiments. Mann-Whitney *U* test was performed to analyze the differences between the samples. *P<0.05, **P<0.01 compared to unstimulated control.

To unravel the mechanism by which IgE engagement of FcεRI on cytokine pre-sensitized B/TSM leads to enhanced CC and CXC chemokine release, we then tested whether IgE alone can induce promoter activity of CCL11, CXCL8, CXCL10 and CCL5 in B/TSM cells. B/TSM cells were transiently transfected with the luciferase reporter constructs driven by respective wild-type chemokine promoters. As shown in Figure 4, IgE stimulation of B/TSM cells significantly enhanced the promoter activity of eotaxin-1/CCL11, 1.42±0.13-fold; IL-8/CXCL-8, 1.46±0.11-fold; IP-10/CXCL10, 1.33±0.05-fold; and RANTES/CCL5, 1.32±0.09-fold, (n = 3 p<0.05) compared to the unstimulated control. Murine isotype mIgG1 (MOPC21) stimulation failed to induce the promoter activity for any of the chemokines tested. Furthermore, as reported earlier in structural cells including ASMC [22], [23], [24], [25], IL-1β stimulation strongly induced the promoter activity of eotaxin-1/CCL11, 2.17±0.17-fold; IL-8/CXCL-8, 3.26±0.30-fold (n = 3, p<0.01); IP-10/CXCL10, 1.65±0.03-fold; and RANTES/CCL5, 1.49±0.02-fold, (n = 3, p<0.05) compared to the unstimulated control (Figure 4). Altogether, these results demonstrate that the IgE induces CC and CXC chemokine gene expression in B/TSM cells by acting at least at the promoter level.

### Lentivirus mediated Syk-silencing abrogates the IgE-induced transcriptional activation of human eotaxin-1/CCL11, IL-8/CXCL8, IP-10/CXCL10, and RANTES/CCL5 promoters in B/TSM cells

**Figure 5:**
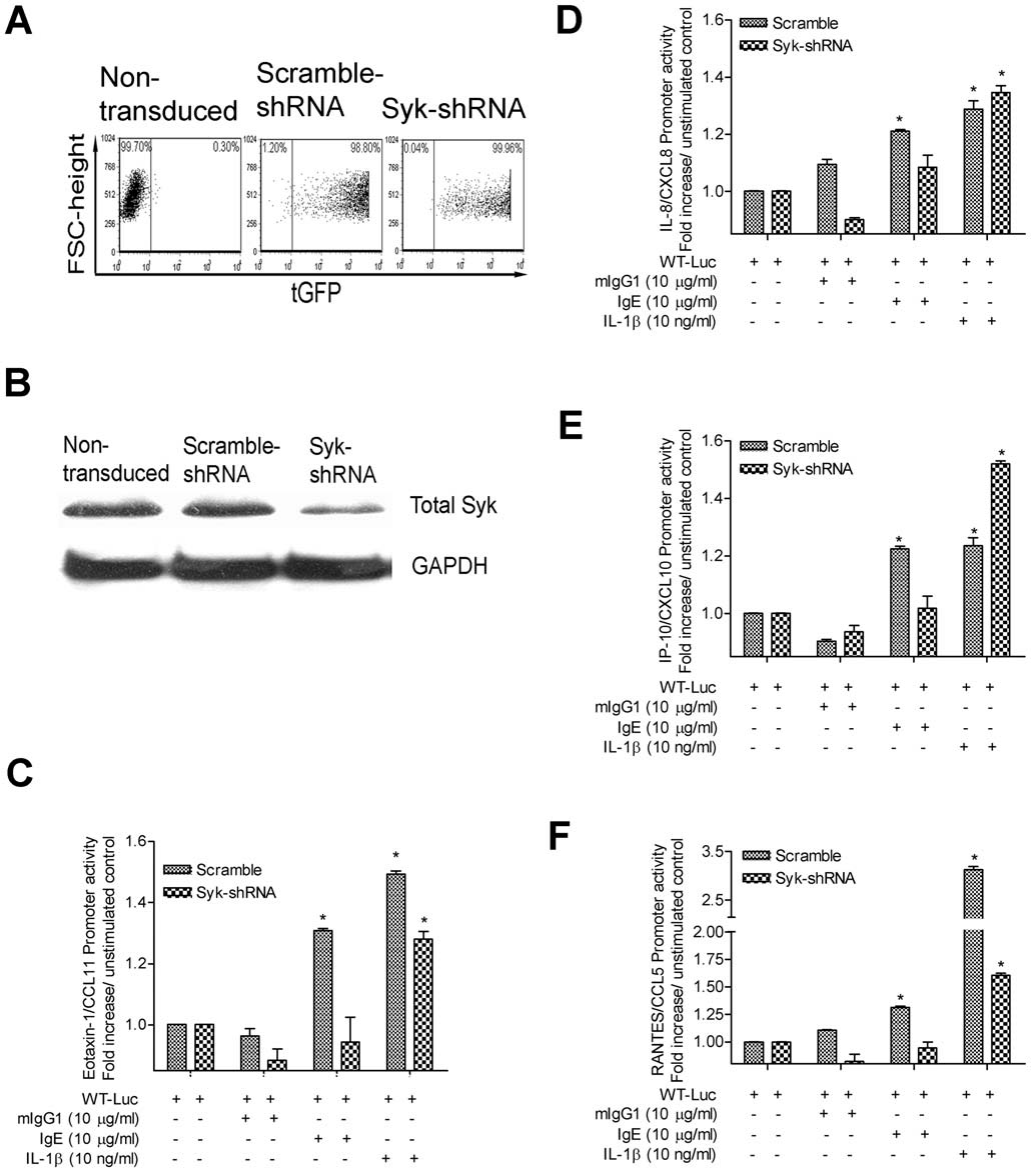
Lentivirus-mediated Syk shutdown abrogates the IgE-induced chemokines promoter activity in human B/TSM cells. Human B/TSM cells were first transduced with a pseudotyped Lentiviral vector expressing specific Syk-shRNA or a non-specific scramble-shRNA. (A) The Lentiviral transduction efficiency was more than 95% for both scramble-shRNA and Syk-shRNA as determined by FACS using the tGFP as the marker for cell sorting. (B) Lentiviral Syk-shRNA transduction in B/TSM cells silenced the Syk expression efficiently as observed by Western blotting. Syk- and scramble-transduced human B/TSM cells were then transiently transfected with luciferase reporter constructs driven by wild-type promoters for human (C) eotaxin-1/CCL11, (D) IL-8/CXCL8, (E) IP-10/CXCL10, or (F) RANTES/CCL5; stimulated with mIgG1-MOPC21, IgE, or IL-1β for 12 h; and respective chemokine promoter activity was measured as mentioned in *Materials and Methods*. Data were normalized according to the *Renilla* luciferase activity, and presented as fold-increase over unstimulated control. Data represents the mean±SD of three separate experiments. Mann-Whitney *U* test was performed to analyze the differences between the samples. *P<0.05, **P<0.01 compared to unstimulated control.

In the light of the above findings, we further investigated whether IgE-induced chemokine expression involved the FcεRI activation. In inflammatory cells such as mast cells, FcεRI activation triggers many signaling pathways including the phosphorylation of FcεRI-β and -γ by Lyn kinase, followed by the activation of Syk through its recruitment to FcεRI [7]. Activation of Syk is crucial for FcεRI downstream signals propagation including phosphorylation of phospholipase Cγ, calcium mobilization, degranulation, and proinflammatory cytokine/chemokine release [7], [26]. These observations suggest that blocking/silencing the Syk expression might be a useful strategy to investigate the FcεRI activation in B/TSM cells. To inhibit the Syk expression, B/TSM cells were transduced with a pseudotyped lentiviral vector expressing specific Syk-shRNA. Mock and scramble sequence were used as negative controls. As shown in Figure 5A, more than 95% of the lentivirus-transduced cells were tGFP positive by FACS analysis. Transduction of cells with Syk-shRNA clone resulted in a highly significant and reproducible decrease in Syk expression, as shown by Western blotting (Figure 5B). However, transduction with the control scramble shRNA failed to reduce Syk expression in B/TSM cells (Figure 5B). To determine if the transcriptional activation of chemokine expression by IgE is affected in the absence of Syk, stably Syk-silenced B/TSM cells were transiently transfected with wild-type promoters for earlier studied CC and CXC chemokines and stimulated with IgE, IL-1β, mIgG1 or left unstimulated. As shown in Figure 5C, D, E and F, IgE-induced chemokines promoter activity over unstimulated control was completely abrogated (eotaxin-1/CCL11, 1.30±0.01 to 0.94±0.14-fold; IL-8/CXCL8, 1.21±0.01 to 1.08±0.07-fold; IP-10/CXCL10, 1.22±0.01 to 1.01±0.07-fold, and RANTES/CCL5, 1.31±0.02 to 0.94±0.09-fold) in Syk-shRNA-transduced compared to the scramble-shRNA-transduced B/TSM cells. However, in contrast, lentivirus-mediated Syk-silencing in B/TSM cells did not affect the IL-1β-induced promoter activity for IL-8/CXCL8 and IP-10/CXCL10 (Figure 5D and E). Moreover, although eotaxin-1/CCL11 and RANTES/CCL5 promoter activity was slightly decreased, it was still significant compared to the unstimulated control in IL-1β-stimulated Syk-silenced B/TSM cells (Figure 5C and F). The isotype mIgG1 (MOPC21) stimulation did not affect the promoter activity for any of the chemokines in either Syk-shRNA or scramble-shRNA–transduced B/TSM cells (Figure 5). Taken together, these results suggest that the IgE-sensitization of B/TSM cells induce the chemokine expression at least via involving Syk activity and the respective chemokine promoter activation.

## Discussion

Several studies have investigated the regulation of high affinity IgE receptor (FcεRI) expression in inflammatory cells both *in vivo* and *in vitro*, and a correlation has been established between the serum IgE levels and the FcεRI expression on cell surface [28], [29]. However, IgE is not the only factor governing FcεRI expression, since mast cells from IgE-deficient (IgE^−/−^) mice express low levels of FcεRI [30]. Therefore, it is indeed apparent that the basal FcεRI expression is under the control of some other regulatory mechanisms. Moreover, although the ASM cells were recently shown to respond to IgE through the expression of a tetrameric FcεRI complex (αβγ2) [15], factors that modulate the FcεRI expression by ASM remain unknown.

In the present study, we demonstrated that proinflammatory (TNF-α) and Th2 (IL-4) cytokines upregulate the FcεRI-α expression in cultured human B/TSM cells both at transcript and protein level. This was strikingly a novel finding as despite being central proinflammatory mediators in allergic inflammation [10], [11], [12], [13], TNF-α and IL-1β have not previously been investigated in the context of FcεRI modulation in any cell type. Interestingly, IgE engagement of FcεRI on TNF-α pre-sensitized B/TSM cells significantly augmented the eotaxin-1/CCL11, IL-8/CXCL8, IP-10/CXCL10, and RANTES/CCL5 but not TARC/CCL17 release. In addition, IgE sensitization of B/TSM cells induced the gene expression for eotaxin-1/CCL11, IL-8/CXCL8, IP-10/CXCL10, and RANTES/CCL5 at promoter level, which was completely abrogated upon Lentivirus-mediated Syk silencing. Our data underline the fact that proinflammatory and Th2 cytokine-induced FcεRI regulation in B/TSM cells could significantly contribute to the airway inflammation via potentially novel mechanisms involving IgE/FcεRI complex.

TNF-α is a prototype proinflammatory cytokine which has been proposed to exert deleterious effects directly on airway smooth muscle, including its most recently explored roles in (i) activation of transient receptor potential channel 3 (TRPC3) leading to abnormal store-operated calcium influx, and (ii) upregulation of CD38 which regulates intracellular calcium and plays a role in airway hyperresponsiveness [31], [32]. Both TNF-α and IL-1β, independently, are known to induce IL-8/CXCL8 and RANTES/CCL5 release in ASM [33]. IL-8/CXCL8 is a chemoattractant for neutrophils, eosinophils, and to a lesser extent, T-lymphocytes, while RANTES/CCL5 acts as a chemoattractant for T-cell, eosinophils and monocytes. TNF-α also induced the release of IP-10/CXCL-10, a potent chemokine for activated T cells, NK cells and mast cells; and its expression is differentially modulated by vitamin D in human ASM cells [34]. In the present study, TNF-α stimulation augmented both FcεRI-α transcript and protein expression in B/TSM cells. Of note, we did not incubate the cultured B/TSM cells with cytokines and IgE simultaneously as it could mask the effect of IgE. Indeed, we first pre-sensitized the B/TSM cells with cytokines and then engaged the receptor with IgE stimulation. Interestingly, TNF-α pre-sensitization (and thus FcεRI upregulation) followed by IgE stimulation augmented the selective eotaxin-1/CCL11, IL-8/CXCL8, IP-10/CXCL10, and RANTES/CCL5 chemokines release, compared to IgE non-stimulated control. This was quite a plausible observation at functional level since IgE could engage the TNF-upregulated FcεRI by a higher magnitude than basal FcεRI expressed on B/TSM cells. Surprisingly, IL-1β enhanced the FcεRI-α mRNA expression and had slight but not significant effect on FcεRI-α protein upregulation. In accordance with this, IgE stimulation following IL-1β pre-sensitization did not augment the chemokine release compared to IgE-unstimulated cells. This may be explained by the involvement of post-transcriptional regulatory mechanisms in FcεRI protein expression in response to IL-1β stimulation, and thus low magnitude of IgE-mediated signaling. Taken together, our observations essentially provide another mechanism by which TNF-α contribute to airway inflammation, mainly amplifying the FcεRI-mediated ASM activation, and ultimately recruiting inflammatory cells through multiple chemokine expression in airways.

FcεRI-γ chain was shown to be downregulated alongwith FcεRI-α chain by TGF-β1 in bone marrow derived mast cells (BMMC) [35], and has been demonstrated to be the limiting factor governing FcεRIα surface expression in dendritic cells (DCs) [36]. However, we found that only FcεRI-γ transcript but not protein was upregulated by TNF-α. Lack of FcεRI-γ protein upregulation by TNF, IL-1β or IL-4 may suggest some post-transcriptional regulatory mechanisms modulating the FcεRI-γ protein translation. Since B/TSM express all FcεRI subunits (αβγ2) [15], possibility of different regulatory mechanisms in B/TSM FcεRI than trimeric DC (αγ2) [36] cannot be denied. Moreover, since FcεRI-γ chain is also shared by Fc gamma receptor subtypes expressed by B/TSM [37], additional regulatory mechanisms may be in force in controlling FcεRI-γ expression. Detailed studies are therefore required to delineate the regulation and role of FcRγ in amplification or stabilization, if any, of FcεRI surface expression in B/TSM cells.

IL-4 on the other hand, is a prototype Th2 cytokine which in combination with IL-13, induce the class switch-recombination from IgG to IgE by B-cells [6]. IL-4 plays a critical role in atopic diseases and is also known to cause a marked increase in eotaxin-1/CCL11 release in ASM cells [38], [39]. In our present study, interestingly, we found that IL-4 significantly enhances the surface and intracellular FcεRI-α protein expression in B/TSM cells. This was quite interesting as there is a steady-state FcεRI-α mRNA expression under IL-4 stimulation, suggesting the coherent translation of FcεRI-α transcript into the protein at 24 h, 72 h after stimulation (Figure 1B, 2A and B). Our data, therefore, strongly supports the previously observed positive role of IL-4 in the transcription of FcεRIα-chain in human mast cells, eosinophils from atopic dermatitis patients, human dendritic cells, and human neutrophils [8], [40], [41], [42].

Classical paradigm entails the IgE binding to FcεRI as a ‘passive sensitization’ step in the mast cell activation and requires the multivalent antigens for cross-linking of FcεRI-bound IgE [7]. However, recent reports highlight the IgE-mediated spectrum of effects including the pro-survival effects on mast cells, monocytes, and asthmatic neutrophils through binding to FcεRI [43], [44], [45], [46]. Moreover, IgE alone (i.e. sensitization) induced the expression of multiple cytokines (e.g. IL-6, TNF-α, IL-4 and IL-13) and activated signaling pathways by phosphorylation of several kinases such as Erks, p38, JNK and PKB in normal murine BMMC [44]. In this line, after exploring the FcεRI regulation by proinflammatory and Th-2 cytokines and subsequent IgE-mediated B/TSM activation, we tested our hypothesis of effect of IgE sensitization alone on ASM synthetic function. Interestingly, we found that IgE sensitization induces multiple, selective CC (eotaxin-1/CCL11, RANTES/CCL5) and CXC (IL-8/CXCL8, IP-10/CXCL10) chemokines expression in B/TSM cells at least at promoter level.

In inflammatory cells, activation of Syk is crucial for IgE cross-linking-induced FcεRI downstream signals propagation including phosphorylation of phospholipase Cγ, calcium mobilization, and degranulation [7], [26]. Although details still remain to be investigated, the initial signaling events in non cross-linking model (i.e. IgE sensitization alone) of FcεRI activation include the activation of Lyn and Syk leading to the activation of ERK in mast cells [47], [48]. Interestingly, in our study, lentivirus-mediated Syk shutdown in B/TSM cells completely abrogated the CC and CXC chemokine promoter activity, suggesting the involvement and requirement of Syk activity in IgE sensitization-induced chemokine expression. These results essentially provide a proof-of-principle and increase our understanding that at least initial signaling events of IgE sensitization-induced FcεRI activation are similar, if not the same, in ASM and mast cells. Altogether, our data suggest that blocking or silencing Syk, among others, could present a useful therapeutic strategy for allergic inflammatory disorders.

Collectively, our data demonstrate that proinflammatory (TNF-α) and Th2 (IL-4) cytokines modulate the FcεRI expression in B/TSM, which can further augment the selective CC and CXC chemokine release following IgE exposure. Moreover, IgE sensitization of B/TSM cells induces multiple chemokine gene expression, via involving at least Syk activity and the respective gene promoter activation. Our data, therefore, highlight the fact that proinflammatory and Th2 cytokines directly or indirectly manifest the allergic inflammation, also by regulating FcεRI expression, hence amplifying the IgE/FcεRI associated reactions.
